# A real-time voice cloning system with multiple algorithms for speech quality improvement

**DOI:** 10.1371/journal.pone.0283440

**Published:** 2023-04-03

**Authors:** Weixin Hu, Xianyou Zhu

**Affiliations:** College of Computer Science and Technology, Hengyang Normal University, Hengyang City, China; Menoufia University, EGYPT

## Abstract

With the development of computer technology, speech synthesis techniques are becoming increasingly sophisticated. Speech cloning can be performed as a subtask of speech synthesis technology by using deep learning techniques to extract acoustic information from human voices and combine it with text to output a natural human voice. However, traditional speech cloning technology still has certain limitations; excessively large text inputs cannot be adequately processed, and the synthesized audio may include noise artifacts like breaks and unclear phrases. In this study, we add a text determination module to a synthesizer module to process words the model has not included. The original model uses fuzzy pronunciation for such words, which is not only meaningless but also affects the entire sentence. Thus, we improve the model by splitting the letters and pronouncing them separately. Finally, we also improved the preprocessing and waveform conversion modules of the synthesizer. We replace the pre-net module of the synthesizer and use an upgraded noise reduction algorithm combined with the SV2TTS framework to achieve a system with superior speech synthesis performance. Here, we focus on improving the performance of the synthesizer module to achieve higher-quality speech synthesis audio output.

## Introduction

Speech synthesis originated from text-to-speech (TTS) [1]. The framework of the TTS modeling is mainly divided into text analysis, acoustic model, and vocoder. On the basis of PixelRNN [2] and PixelCNN [3], the WaveNet model is developed as a speech backend processing module. The WaveNet model is widely mentioned in the literature [4]. However, WaveNet is not an end-to-end model; it is an efficient waveform generation model based on deep learning, including gating mechanism and residual connections. In particular, WaveNet only replaces the backend part of the traditional TTS pipeline [5]. Other effective models have also been proposed, such as Deepvoice, but neither of these approaches achieves complete end-to-end speech processing. In 2017, Google reached a major milestone in speech synthesis, developing the first end-to-end speech model called Tacotron [6]. The improved Tacotron2 consists of two parts: a synthesizer and a modified algorithm of the WaveNet model [7]. By referring to the WaveNet model, the naturalness of the generated speech can be significantly improved, and some people even think that the output speech sounds more natural than the human voice. However, this system still has some limitations, not the least of which is its very limited speed.

In response to this problem, some researchers proposed a new model called Fastspeech with reference to TransformerTTS [8]. It greatly improves the synthesis speed of audio while ensuring a certain quality of audio. Then, some researchers proposed the Fastspeech2 model, introducing more inputs to control the quality of synthesized audio [9]. After solving the problem of synthesis speed, researchers began to explore the naturalness of synthesized audio.

To bridge the gap between synthetic audio and natural human voice, in recent years, a new framework based on Tacotron2 has been proposed, called SV2TTS. This framework makes use of most of the components of Tacotron but uses GE2E loss and WaveNet models. This allows the framework to extract a speaker’s voice features for speech synthesis work in less than 5 seconds. SV2TTS has a significant advantage in extracting speaker features. Once it has extracted enough features, the method can produce more effective audio results based on the input text. This audio sounds like the speaker’s voice. Some researchers have proposed replacing the WaveNet model with a WaveRNN-like model to achieve faster and better speech synthesis [10].

SV2TTS performs a different task compared to Tacotron2. The task of Tacotron series focuses more on text-to-speech. In contrast, SV2TTS provides new insights into speech cloning. Thus, SV2TTS replaces the text analysis module of Tacotron2 with a speaker encoder module. Among the three modules of SV2TTS, the speaker encoder comprises a newly created function module that can extract speaker features, which can be added to the synthesizer operations. The other two modules are similar to those of Tacotron2. SV2TTS is a crucial development in the field of natural language processing, which opens up a completely new task for natural language processing and is currently a new target for most researchers in the field of natural speech processing. Research on its three modules has also been performed gradually.

The result of this speech synthesis can determine the value of the desired embedding based on the clusters in the embedding space. However, this also has some limitations. Some researchers have constructed a speech-cloning framework by imitating the SV2TTS framework, but the model still involves some defects, especially with waveform conversion [11]. In this study, we address these defects by constructing a new acoustic model, adding two new algorithms, and implementing a noise reduction function. The results of an experimental evaluation are provided to show that the proposed method achieved superior performance compared to existing models.

The main contributions of this work are summarized as follows.

1. We propose a new algorithmic structure to process long sentences in speech cloning systems.
2. We also provide a pronunciation algorithm structure for homemade words.
3. We also improve existing methods by replacing the preprocessing network module in the synthesizer.
4. Our proposed approach enhances the audio quality of voice clones using a new noise reduction model combining multiple algorithms.

Voice cloning is an emerging technology with a wide range of application scenarios. Voice cloning technology can significantly reduce the cost of media applications such as audiobooks, as well as film and television dubbing. Naturally, its key significance lies in facilitating a sense of anthropomorphic presence in human-computer interaction and advancing technological development in the field of natural language processing. Given the current high level of interest in the concept of metaverses, speech cloning technology can also fill gaps in existing metaverse concepts. In this study, we consider speech-cloning technology with the associated ethical issues in mind. We hope that our research can contribute to the development of new directions in natural language processing. The remainder of this study is organized as follows. First, we briefly review the relevant literature. Then, we discuss the proposed approach in terms of the new acoustic models, algorithms, and denoising functions provided. Finally, we present the experimental results and related analyses and suggest possible avenues for further research.

## Related work

In 1939, Bell Labs introduced an electronic microphone called VODER, which used electronics to simulate the sound resonance. Subsequently, the lab began to explore the possibility of developing a machine to simulate the human voice. By 2009, methods to synthesize the human voice mechanically were already being explored. However, these methods did not evolve owing to the lack of computational technology at the time [12]. Later, some researchers also explored the use of musical instruments to simulate the human voice. Nevertheless, the results were not satisfactory [13].

The first wave of scientific research on speech synthesis was driven by early speech synthesis techniques. These can be divided into three main approaches, including articulation [14, 15], resonant peak [16], and tandem synthesis methods [17]. Among these, the tandem synthesis methods include the PSOLA-synchronous waveform overlay technique of fundamental tones and CHATR-speech splicing based on large-scale speech libraries. The idea of the vocal synthesis method is to simulate the human voice by exploring the human vocal structure. However, the quality of the speech synthesized was not ideal due to realistic factors. The resonant peak synthesis method is based on linguistic rules to simulate as closely as possible the resonant peak structure and other spectral characteristics of human speech [11]. However, the quality of the final synthesized speech leaves much to be desired. The tandem synthesis method, often referred to as the splicing method, is based on the principle of retrieving a large amount of speech audio from a database and then splicing the relevant retrieved audio through some tandem methods to form a new speech waveform. This approach has the advantage of a more natural sound and the disadvantage that the database must store a large number of human voice samples, which is unrealistic.

All three methods mentioned above have certain limitations; thus, models based on innovative ideas of speech synthesis have been developed; for example, the statistical parametric speech synthesis method (SPSS) was created [18]. The main idea is that before the synthesis phase of the model begins, the relevant acoustic parameters are generated by some methods, which are needed for the subsequent phases. The SPSS comprises three main modules in SPSS: text analysis, acoustic, and vocoder modules, which are respectively designed to analyze the input text data, train speech features and parameters, and synthesize speech, respectively. This idea provided a basis for subsequent innovation, among which an acoustic model based on a hidden Markov model (HMM) has exhibited good results [19]. Compared with previous methods, SPSS has several advantages; on the one hand, it generates more natural and flexible audio, making it easy to modify parameters to control speech generation. On the other, it requires less costly data and fewer recordings than tandem synthesis. However, SPSS also has the disadvantage that the generated speech has low intelligibility due to artifacts, such as murmurs, hums, or noisy audio, and the generated voice is still robotically produced and can be easily distinguished from human-recorded speech.

With the development of deep learning, various models have begun to emerge. In particular, neural networks are being more widely adopted for speech synthesis.

The first and most notable example is the Tacotron model, which is the first end-to-end TTS neural network model that only needs to input the corresponding text sequence to generate the associated Mel-spectrogram. Subsequently, the waveform is generated using the Griffin-Lim algorithm. Moreover, a more natural result can be obtained using the CBHG module and sequence encoder and decoder. Tacotron2 uses the WaveNet model to improve the previous generation of products. The final Griffin-Lim algorithm is replaced by a WaveNet-like algorithm that improves the naturalness of the generated speech. At the same time, Tacotron2 does not use the CBHG module but uses the traditional LSTM and related convolutional layers and adds some post-processing. After Tacotron and Tacotron2 were published, researchers began to adjust and build new models based on these methods to pursue better experimental results, such as ClariNet [20], FastSpeech 2s [21], and EATS [22]. SV2TTS is an improvement of Tacotron2 that does not modify the Tacotron2 model structurally but changes the vocoder part. Thus, SV2TTS is almost identical to Tacotron2 in the synthesizer encoder principle [23].

Similar to Tacotron2, in SV2TTS, the speech synthesis process is divided into three parts: speaker encoder, synthesizer encoder, and vocoder encoder. It extracts speaker features through a speaker encoder and outputs a melt spectrum through a synthesizer encoder. Finally, SV2TTS uses a vocoder encoder to output the frequency-domain spectrum as a time-domain waveform. Below, we introduce the relevant modules of SV2TTS.

The speaker encoder is composed of a 40-channel log Mel-spectrogram. The speaker’s voice features are captured through recursive LSTM layers, and the final output is normalized. In this case, the three LSTM layers are connected unidirectionally, and the output of each layer is the input of another layer. In the system, this module is relatively smaller than the other two and implements a simpler load work. When the 40-channel log Mel-spectrogram passes through this module, a 256-element vector is outputted.

Then, the speaker’s embedding information captured by the speaker encoder is combined with the input text information in the synthesizer to generate the Mel-spectrogram through the same network architecture as Tacotron2. In this case, the text input synthesizer module is combined with the output of the speaker encoder to form a tandem vector after vector conversion and a three-layer convolution network followed by a bidirectional LSTM. Subsequently, an attention mechanism with two unidirectional LSTM layers is projected into the Mel-spectrogram, which is then summed by a residual to output the Mel-spectrogram. In the proposed approach, a text determination module is added between the text and the embedded information to improve the pronunciation of speech clones on non-standard words.

Finally, the Mel-spectrogram is generated into a waveform by the WaveNet model in the vocoder part. In SV2TTS, the vocoder part relies on the WaveNet model, which is the same as Tacotron2. The main contribution of the proposed model is in the vocoder part. Here, we use the WaveRNN model as the main structure of the vocoder and add a noise reduction function to improve the effectiveness of the speech synthesis.

## Framework and principles

**Fig 1:**
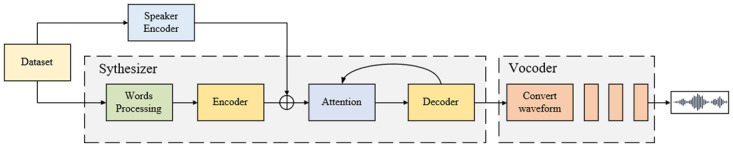
Overall structural framework.

The overall structural framework of this paper is shown in Fig 1. Here, we adopt the traditional speech cloning structure: speaker encoder, synthesizer, and vocoder. In the training process, we prioritize the speaker encoder, extract speaker embeddings, and add relevant embeddings in the synthesizer training process. Moreover, we process the vocabulary during the synthesis process so that the entire system can handle more complex words and phrases. The primary function of the vocoder is to convert the melodic spectrogram into a waveform and output it as audio. Finally, we use a multi-algorithmic audio noise reduction to improve the audio quality.

### Structural framework

**Fig 2:**
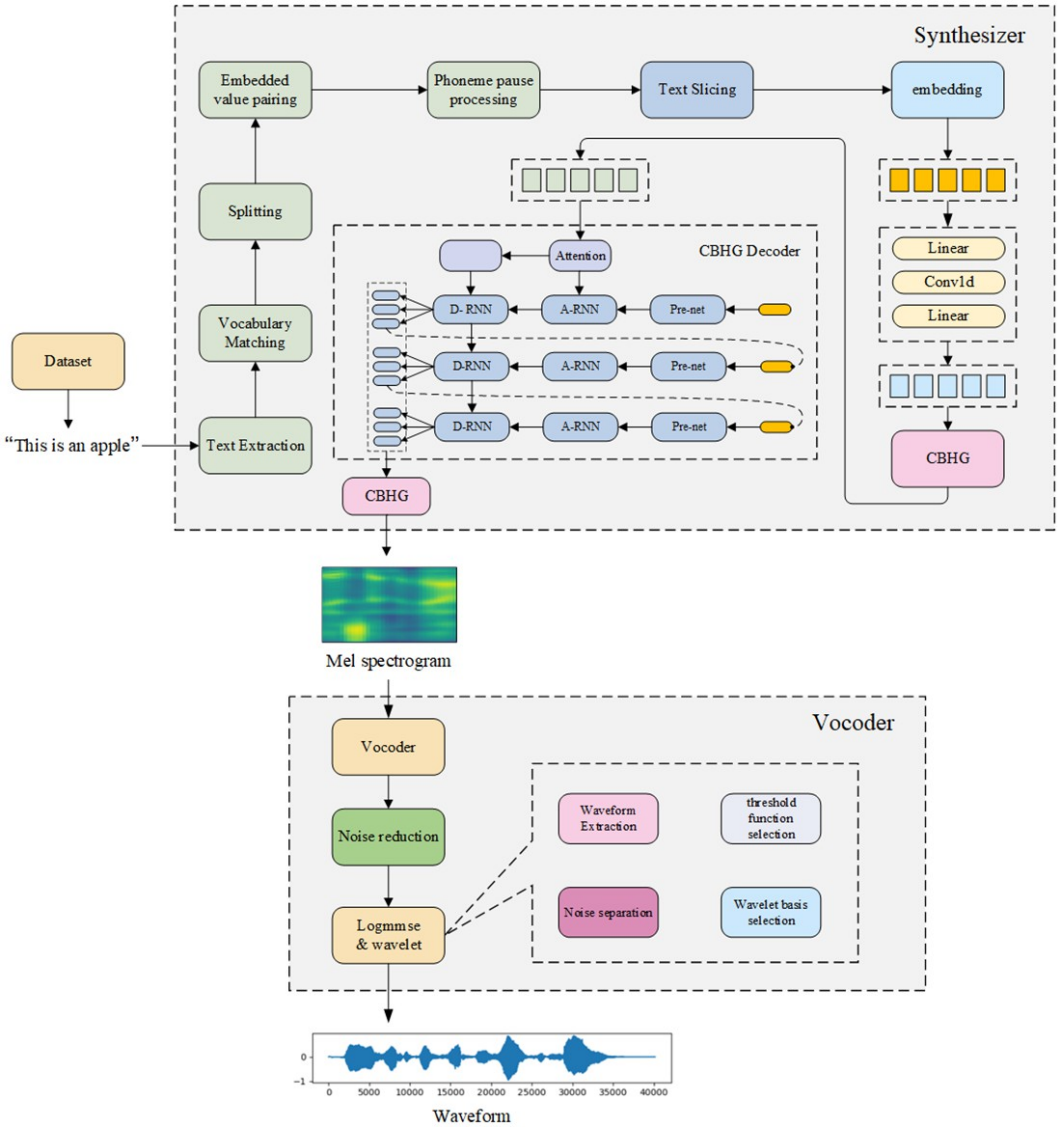
Voice basic structure.

The core infrastructure is shown in Fig 2. The input audio is extracted using the acoustic features, and the speech synthesis is performed within the synthesizer module. Finally, the waveform is outputted by the vocoder. We also include some improvements to the vocoder module. In this framework, WaveRNN can improve the synthesis speed of the waveform effectively. Its basic principle is as follows:

$$\begin{array}{c} T(x)=|x|\sum_{i=1}^{N}(\alpha (op_{i})+\beta (op_{i})) \end{array}$$ (1)

where *x* is the number of samples, *α*(*op*_*i*_) is the time required to compute at layer *i*, and *β*(*op*_*i*_) is the computational overhead at layer *i*. This formula allows the model to form several samples using short audio files.

### Synthesizer module improvements

In this study, we propose a method to overcome the limitations of existing synthesizer modules in terms of long sentences and extra-lexicon processing and improve the overall framework.

**Fig 3:**
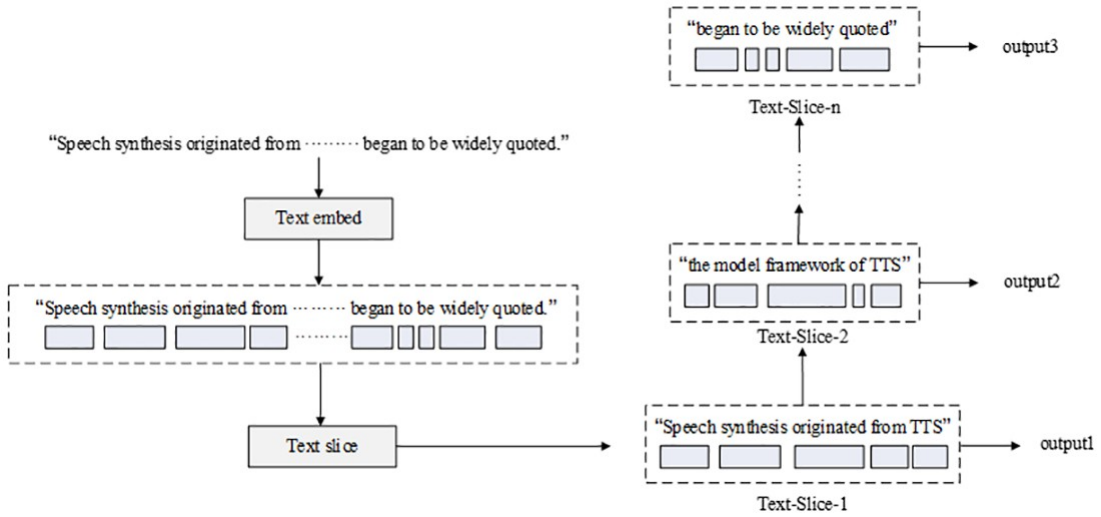
Text slicing.

Existing speech cloning models involve two flaws that significantly affect the effectiveness of speech synthesis. The first is the processing of long sentences; owing to the limited function of the attention mechanism, the synthesis of long sentences deteriorates the further the sentences are processed. The second flaw is that the speech synthesis result is accompanied by sound artifacts like murmurs, affecting the listening experience. As shown in Fig 3, we propose a novel algorithmic improvement for long sentence processing in the synthesizer module by slicing the sentences into shorter components.

In this study, a text extraction module is added to the synthesizer module to match and compare the extracted text with the vocabulary list, extract and split the non-standard words separately, and then backfill them and perform embedding value matching and phoneme stopping on the modified text. After the text vector is combined with the embedded values, it enters the convolutional network of the synthesizer for processing.

In the synthesizer module, the original project used a preprocessing model for the character embedding vectors. The original pre-net network module consists of a neural network module with multiple superimposed fully connected layers and a dropout parameter after each fully connected layer to prevent overfitting. In this study, we replace the preprocessing module with a fully-connected-one-dimensional convolutional network module. The fully connected layer improves the generalization of the network structure but also introduces certain limitations. The improved generalization capability also requires passing a large number of parameters, significantly reducing the computational speed. Adding a convolutional layer then accelerates the computational speed of the model, and the probability of overfitting is further reduced by taking advantage of the ability of the convolutional layers to process a large number of parameters efficiently.

### Noise reduction treatment

In this study, we propose a new complex noise-reduction module integrated into the vocoder module to improve the quality of speech synthesis. In the new noise reduction model, we fully use advantages of multiple algorithms and combine multiple noise reduction algorithms to improve the quality of speech systematically. Many traditional speech algorithms have been developed, such as Wiener filtering and various speech enhancement algorithms. The log-MMSE algorithm used above is computationally intensive. Nevertheless, it is also more efficient. The experimental results have shown that it removed noise effectively.

In this algorithm, we assume that the signal with noise in the time domain is *y*(*n*) = *x*(*n*) + *p*(*n*), where *x*(*n*) is a pure and noiseless signal, and *p*(*n*) is a noisy signal. The formula changes after transferring to the frequency domain, *Y*(*ε*) = *X*(*ε*)+ *P*(*ε*). The optimal log MMSE estimator derived in this study is shown in Eq (2).

$$\begin{array}{c} \hat{X_{b}}=\frac{\eta_{b}}{\eta_{b}+1}exp\{\frac{1}{2}\int_{V_{k}}^{\infty }\frac{e^{-t}}{t}dt\}Y_{B}=G_{LSA}(\eta_{b},V_{b})Y_{B} \end{array}$$ (2)

In this formula, $V_{b}=\frac{\eta_{b}}{\eta_{b}+1}\gamma_{b}$.

The function uses a priori SNR *η*_*b*_ and a posteriori SNR *γ*_*b*_ composes of *G*_*LSA*_(*η*_*b*_, *V*_*b*_).

Among these, $\eta_{b}=\frac{\lambda_{x}({Y_{b}}^{2})}{\lambda_{d}(b)}$ $\gamma_{b}=\frac{{Y_{b}}^{2}}{\lambda_{d}(b)}$

λ_*x*_(*b*) = *E*{|*X* (*ε*_*b*_)|^2^} is the variance of the *b*th spectral component of the pure signal *X*(*ε*).

Moreover, λ_*d*_(*b*) = *E*{|*X* (*ε*_*d*_)|^2^} represents the variance of the *b*th spectral component of the noise signal. The other values are deduced from *Y*_*B*_. The final derivation is expressed as Eq (3).

$$\begin{array}{c} \eta_{b}(m)=\alpha \frac{{X_{b}}^{2}(m-1)}{\lambda_{d}(k,m-1)}+(1-\alpha )max[\gamma_{b}(m)-1,0] \end{array}$$ (3)

Although log-MMSE removes noise well, many noises that cannot be separated still remain, implying that the log-MMSE algorithm separating the pure signal and the noisy signal cannot be completely separated. Adding the discrete wavelet transform might be a proper solution for this problem. The discrete wavelet transform can start from the frequency domain instead of the time domain and can separate the specific noise very well.

In the discrete wavelet transform process, we first set a critical threshold λ. This threshold value distinguishes whether the wavelet coefficients are caused by noise or signal. When the coefficients are greater than λ, they are considered to be mainly caused by the signal, and this part needs to be retained. In contrast, if the coefficients are less than λ, they are considered to be caused by the noise and need to be removed. The wavelet coefficients are then subjected to an inverse wavelet transform operation to obtain the wavelet transform-denoised signal.

The implementation of the wavelet transform requires several steps. The first step is selecting the wavelet base: we often try to choose wavelets that simultaneously exhibit orthogonality, high vanishing moment, tight branching, symmetry, and asymmetry. However, this is not always possible because only Haar wavelets are symmetric and asymmetric simultaneously. Moreover, high vanishing moments and tight branching are contradictory and cannot exist simultaneously. Therefore, the choice of the appropriate wavelet to use in a given application must rely on the characteristics of the signal, and, as a rule, wavelets with tight branches are the most typical choice. Second, the choice of the threshold value directly impacts the denoising effect. Third, the choice of the threshold function is also crucial; different inverse functions reflect different strategies for processing the wavelet coefficients, and it is a rule for correcting the wavelet coefficients. In the threshold selection process, we use the traditional wavelet transform threshold selection method; the absolute value of each element in the signal *x*(*i*) is sorted in ascending order and squared to obtain the sequence *F*(*s*) as shown in Eq (4).

$$\begin{array}{c} F(s)={\left(sort(|x|)\right)}^{2}\;\;\;\;\;\;\;\;\;(s=0,1,2,\ldots ,N-1)\;. \end{array}$$ (4)

Suppose that the square root of the sth element of *F*(*s*) is expressed as shown in Eq (5).

$$\begin{array}{c} \delta_{s}=\sqrt{F(s)}\;\;\;\;\;\;\;\;\;(s=0,1,2,\ldots ,N-1) \end{array}$$ (5)

Then, the risk arising from this threshold is shown in Eq (6).

$$\begin{array}{c} risk(s)=[N-2s+\sum_{i=1}^{s}F(i)+(N-s)F(N-s)]/N \end{array}$$ (6)

If the value of *s* corresponding to the minimum risk point is taken according to the risk function, the threshold function can be defined as in Eq (7).

$$\begin{array}{c} \delta_{s}=\sqrt{F(s_{min})} \end{array}$$ (7)

In this study, *s* is considered the 0th element in the threshold selection for processing. The two most commonly used functions are the hard and soft thresholding functions. In addition, the Garrote function can be considered. This function steps in between the soft and hard thresholding functions. Moving on to the choice of threshold, we first need to confirm the desired denoising effect, which can be determined using the signal-to-noise ratio (SNR) of the signal and the root mean square error (RMSE) of the estimated signal and the original signal. Eq (8) is the basic expression for the wavelet transform after the basic wavelet has been shift transformed.

$$\begin{array}{c} WL(\omega ,\gamma )=\frac{1}{\sqrt{\omega }}\int_{-\infty }^{\infty }f(t)*\psi (\frac{t-\nu }{\omega })dt \end{array}$$ (8)

where the soft threshold function is shown in Eq (9).

$$w_{\varepsilon }=\left\{\begin{array}{cc} \left[sgn\left(w\right)\right]\left(\left|w\right|-\varepsilon \right) & \left|w\right|\geq \varepsilon \\ 0 & \left|w\right|<\varepsilon \end{array}\right.$$ (9)

**Fig 4:**
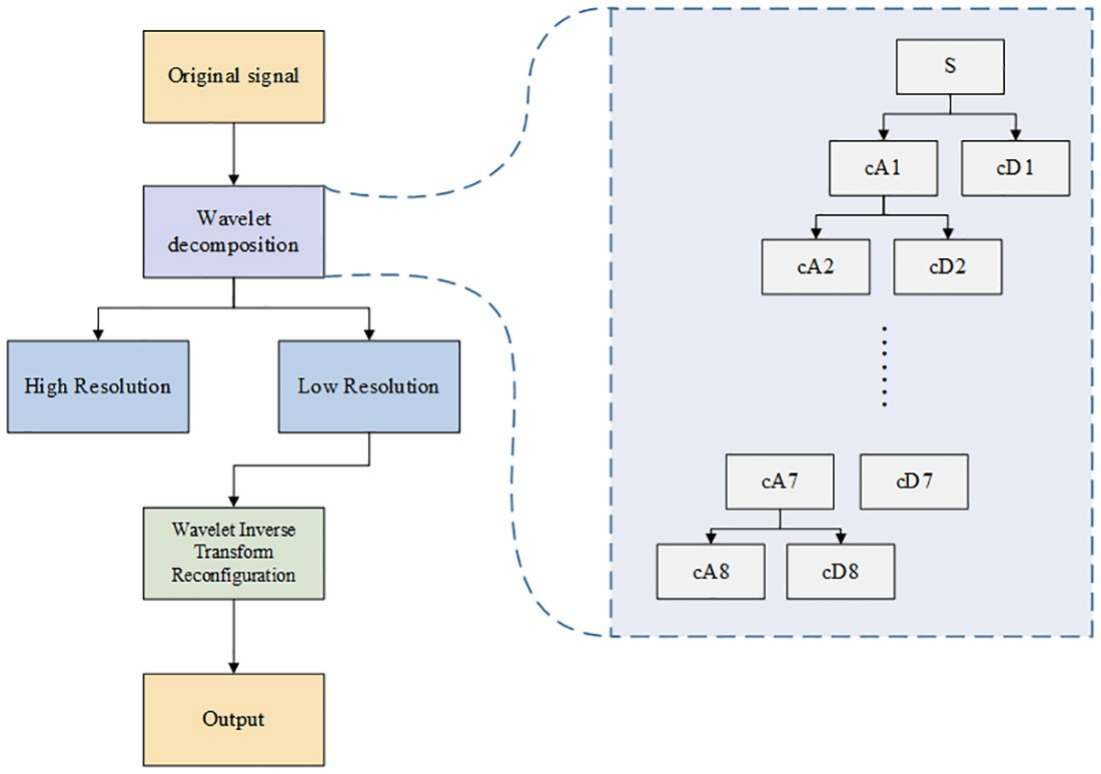
Wavelet structure.

As shown in Fig 4, the wavelet transform denoising process undergoes three steps: wavelet decomposition, signal separation, and wavelet reconstruction. In this study, we selected the d8 parameter for wavelet decomposition operation. In particular, d8 is the 8th-order wavelet tree, which separates the noisy signal from the pure signal more effectively than the d4 parameter. Finally, a purer signal is output after wavelet reconstruction.

In this study, we use the discrete wavelet transform to solve the noise log-MMSE cannot handle. However, adding the wavelet transform also poses certain problems. This is because wavelet transform cuts for a specific threshold of noise can lead to instability of the audio, making the audio break, bursts, distortion, and so forth. Nevertheless, we found that the spectral reduction method takes advantage of the uncorrelated characteristics of additive noise and speech, and can replace the spectrum of noise during the presence of speech with the estimated value of the noise spectrum measured without a speech gap, which can alleviate the problems caused by the wavelet transform effectively.

Spectral subtraction and wavelet transform are similar in that they both deal with audio information in the frequency domain. The main equation is shown in Eq (10), where *F*_*k*_(*x*) is the frequency domain representation of the speech at a specific moment, and D(·) is the noise segment calculation function. A and b are two constants for the over-subtraction and gain compensation factors, respectively.

$$\begin{array}{c} {\left|\hat{F_{k}}\left(x\right)\right|}^{2}=\{\begin{array}{cc} {\left|F_{k}\left(x\right)\right|}^{2} & -a\times D\left(x\right),{\left|F_{k}\left(x\right)\right|}^{2}\geq a\times D\left(x\right)\\ \; & b\times D\left(x\right),{\left|F_{k}\left(x\right)\right|}^{2}<a\times D\left(x\right) \end{array} \end{array}$$ (10)

## Results

We experimented with male and female voices, long and short sentences, extra lexical words, and different voice tones. All experimental results indicated that a higher speech synthesis quality was achieved after adding the new module.

**Fig 5:**
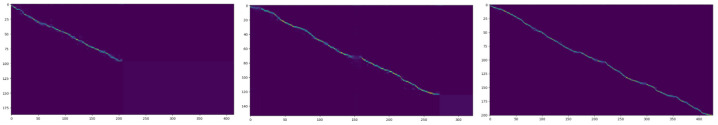
Smooth curve change graph during training.

**Fig 6:**
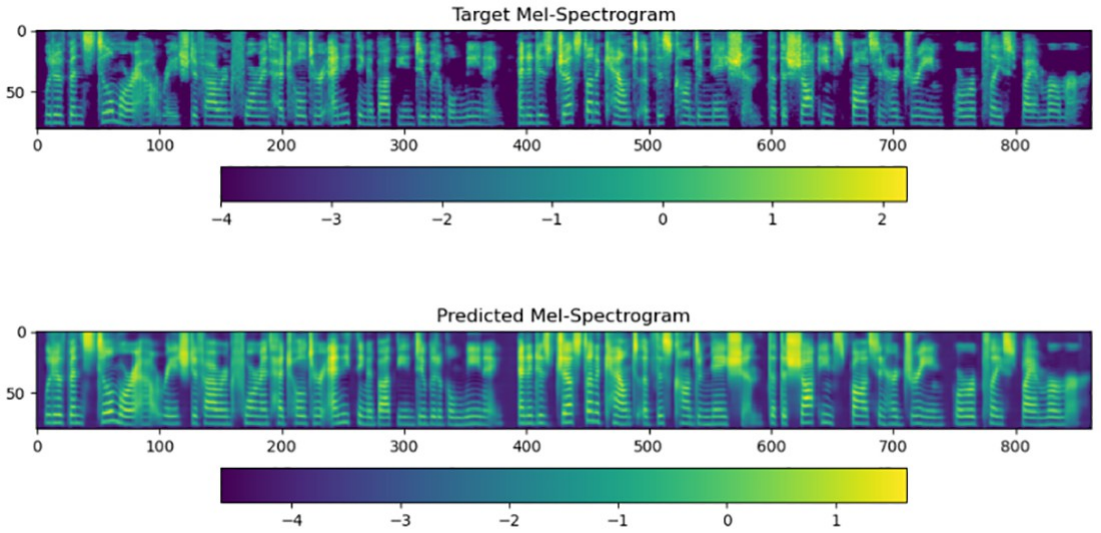
Iterating over 28,500 steps of the Mel-spectrogram.

We experimentally observed the training process of the model and evaluated the model’s performance. Fig 5 is the three stages of the alignment link in the iterative training process of the synthesizer. As shown in Fig 5, the synthesizer is iteratively trained to achieve smooth alignment at a specific number of steps. Fig 6 exemplifies the Mel tone spectrogram quality. When the iterative training reaches step X, the loss value is 0.38270, which has dropped to a satisfactory result. Furthermore The deviation of the predicted spectrum from the actual Mel-spectrogram is minimal, indicating that the model can synthesize speech audio accurately.

**Fig 7:**
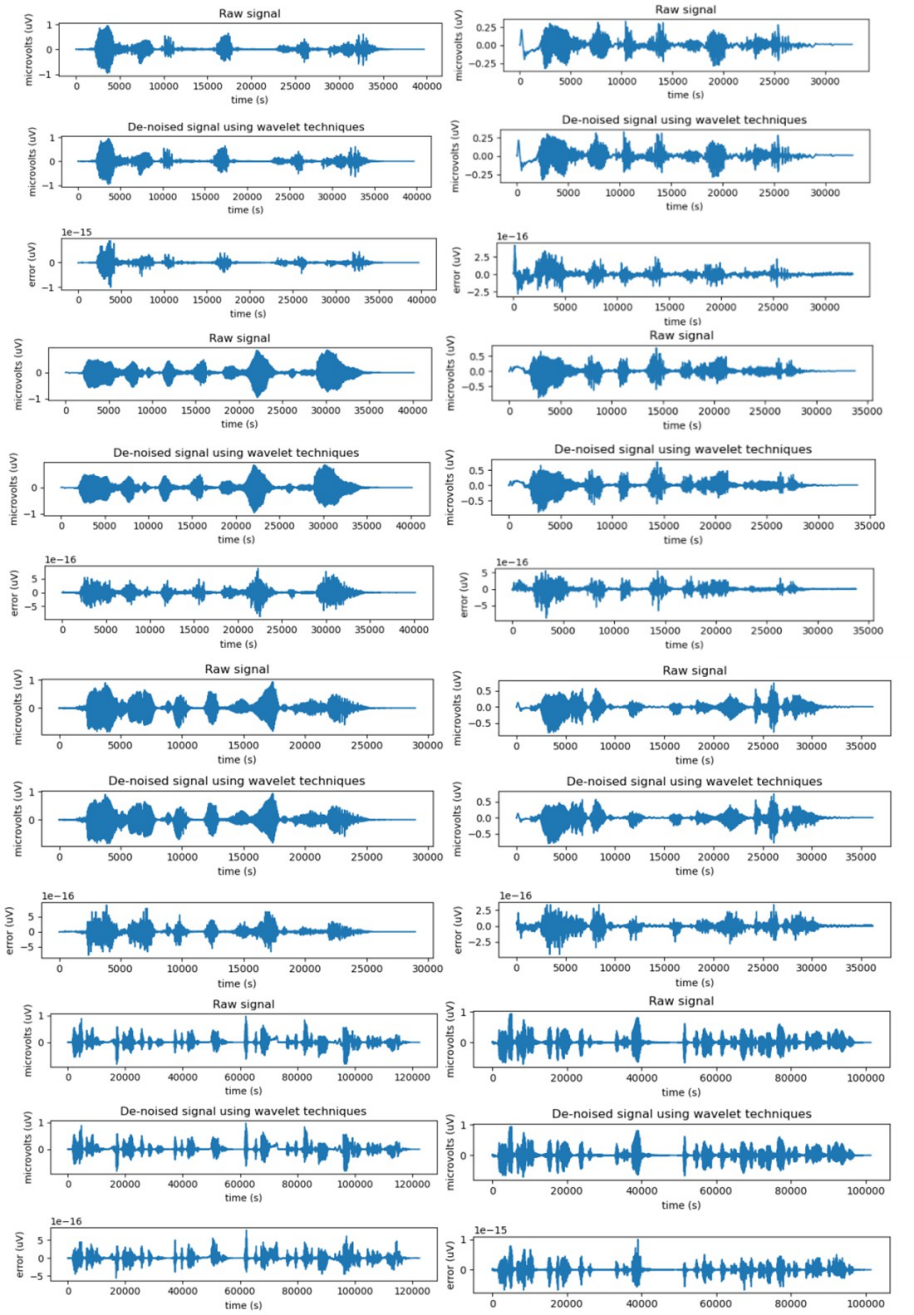
Experimental waveform comparison chart.

As shown in Fig 7, we compared the waveforms of the original audio with those obtained after noise reduction obtained experimentally and plotted separately the noise waveforms, which cannot be clearly visualized in the plotted waveforms because the noise and the human voice waveforms are superimposed. In the experimental process, we compared the original sound with the sound after noise reduction processing in several sets of audio files and measured the audio files in terms of the MOS, PESQ, and CD measures. Table 1 shows that the quality of the processed audio information was significantly improved compared with the audio before processing.

**Table 1:** MOS standard comparison table.

Signal	MOS↑	PESQ↑	CD↑
Row signal	2.12	1.17	8.20
De-noise signal	3.85	3.55	8.93

We also experimented with the processing of long sentences. In particular, we used long sentences, paragraphs, and utterances. By experimenting with sentences of different lengths, the sentence lengths necessary for optimal quality of the output audio were determined. We also experimented with extra-dictionary vocabulary by inputting homemade individual words into the system.

In this study, we implemented a simple speech synthesis system based on the SV2TTS-related module and confirmed its benefits compared to existing methods. We also added a noise reduction module to the system. We used an English dataset for training. Here, we describe the specific procedures of several experiments.

The open-source dataset Librispeech ASR corpus was used in the experiments, which has a large number of pure speech audio files. Due to equipment limitations, we used approximately 1000 audio files for the training dataset. Moreover, we tested different types of audio, including voices from older adults, children, men, women, and celebrities. Roughly 20 testing sets were conducted for each type, using five different audio files as input for each set.

For noise reduction, we conducted several sets of experiments to demonstrate the feasibility of the method. In particular, we conducted ablation experiments using the source output audio as the reference audio. We explored the audio quality scores after using the log-MMSE algorithm alone, the audio quality scores of the log-MMSE algorithm combined with the wavelet transform, and the audio quality scores of the wavelet transform combined with the log-MMSE algorithm for spectral subtraction, respectively. Moreover, several evaluation metrics, such as MOS, SNRseg, WSS, STOI, PESQ, and CD, were used to evaluate various aspects of the audio. Among these, MOS scores the final quality of the audio, SNRseg evaluates the segmental signal-to-noise ratio of different audio, WSS is a weighted spectral skew measure where a smaller score indicates less distortion, STOI evaluates the intelligibility of the speech, PESQ evaluates the quality of the speech more statistically, and CD. Note that the higher the score, the better the performance of the speech. The experimental results are shown in Table 2. We found that the audio quality maintained an improving trend, even though the metrics fluctuated slightly.

**Table 2:** Speech noise reduction ablation experiment table.

Signal	MOS↑	SNRseg↑	WSS↓	STOI↑	PESQ↑	CD↑
log-MMSE	3.04	10.73	37.47	0.95	1.81	8.85
log-MMSE + wavelets	3.34	11.82	24.06	0.96	2.59	8.64
log-MMSE + wavelets (with Spectrum subtraction)	3.85	13.70	15.84	0.99	3.55	8.93

The performance of the proposed model was further evaluated by objectively analyzing the audio quality of the speech synthesized by different models. Moreover, since it is impossible to compare parameters through the corresponding reference audio, we still choose to use MOS indicators and PESQ indicators, which can show the audio quality more intuitively. Table 3 shows that our audio quality is higher than that synthesized with other models. In addition, we measured the CD index. In this index, our model also showed relatively good results. The evaluation results can be seen in Table 3. As shown in Table 3, the comprehensive evaluation of our generated speech audio outperforms other models. The proposed method is superior in fluency, naturalness, and clarity compared with the audio synthesized by other models.

**Table 3:** MOS standard comparison table.

Signal	MOS↑	PESQ↑	CD↑
DeepVoice	2.25	1.78	9.41
Tacotron2	3.45	2.83	7.99
SV2TTS	2.12	1.17	8.20
Fastspeech2	3.52	1.65	8.99
ours	3.85	3.55	8.93

We constructed a system to present our speech synthesis results visually. The features of the speaker’s voice were extracted by inputting multiple short audio files of the same speaker; after obtaining enough rhythmic information, a UMAP graph was generated where data clusters were constructed, and embeddings were selected from the data clusters as acoustic features of the synthesized audio. The speech quality of the synthesized audio was improved by using the noise reduction function module. In this case, the acoustic features in each input audio appeared as clusters in the UMAP visualization view. If the UMAP plot is considered a coordinate system, the acoustic feature points in similar domains indicate the similarity of their acoustic features. The sound spectrum graph of the original and the synthesized audio can also be visualized.

## Conclusion

In this study, we use various algorithms to improve the quality of the audio synthesized by the speech cloning system. Among the improvements performed to the synthesizer, the code structure was improved in terms of long sentence processing. After testing, the long sentences synthesized by the speech cloning system are more natural and less prone to sound blurring. Furthermore, a structure for self-made vocabulary pronunciation was added. This structure allows the speech cloning system to pronounce special words. In addition, the preprocessing structure was improved by introducing a novel feedforward network, trained using a smaller computational cost, improving the training efficiency. Finally, we implemented a multi-algorithm speech-denoising module for noise reduction of the model. From the experimental results, we can see that the multi-algorithm speech-denoising module can improve the audio quality produced by speech cloning, and the complementary advantages of wavelet transform algorithm, log-MMSE algorithm, and spectral subtraction algorithm denoising significantly improved the produced speech quality.

Nevertheless, as a recently developed speech processing subtask, the speech cloning project has room for improvement. After the experiments, we found that although the speech cloning system was relatively well-functioning, some directions are worth studying. First, the training cost of the three modules, including the time cost and the number of datasets, is huge. Therefore, how to use a smaller number of datasets to lightweight the model is a subsequent stream of research. Second, when conducting tests, we found that some synonyms and near-synonyms still have a gap compared with natural human voices after speech cloning. Finally, owing to the excellent performance of the transformer structure, which has been an increasing study topic in recent years for natural language processing tasks, we believe that it can replace the Tacotron structure for better speech cloning tasks.

## References

[1] Taylor P. Text-to-speech synthesis. Cambridge: Cambridge University Press; 2009.

[2] Van Oord A, Kalchbrenner N, Kavukcuoglu K. Pixel recurrent neural networks. In: Balcan MF, Weinberger KQ, editors. ICML’16: Proceedings of the 33rd International Conference on Machine Learning; 2016 Jun 19-24; New York, USA. 48: 1747–1756.

[3] Kalchbrenner N, Espeholt L, Vinyals O, Graves A. Conditional image generation with PixelCNN decoders. Adv Neural Inf Process Syst. 2016;30: 4797–4805.

[4] Oord AV, Dieleman S, Zen H, Simonyan K, Vinyals O, Graves A, et al. WaveNet: A generative model for raw audio. In: Bonafonte A, Prahallad K, editors. SSW9: Proceedings of the 9th ISCA Speech Synthesis Workshop; 2016 Sep 13–15; Sunnyvale, CA, USA. 125: 2.

[5] LeCun Y, Bengio Y, Hinton G. Deep learning. Nature. 2015;521(7553): 436–444. doi: 10.1038/nature14539 26017442

[6] Wang Y, Skerry-Ryan R J, Stanton D, Wu Y, Weiss RJ, Jaitly N, et al. Tacotron: Towards end-to-end speech synthesis. INTERSPEECH 2017: 18th Annual Conference of the International Speech Communication Association; 2017 Aug 20-24; Stockholm, Sweden. 4006–4010.

[7] Shen J, Pang R, Weiss RJ, Schuster M, Jaitly M, Yang Z, et al. Natural TTS synthesis by conditioning Wavenet on MEL spectrogram predictions. Proceedings of the 2018 IEEE International Conference on Acoustics, Speech, and Signal Processing (ICASSP); 2018 Apr 15-20; Calgary, Canada. 4779–4783.

[8] Ren Y, Ruan Y, Tan X, et al. Fastspeech: Fast, robust and controllable text to speech. Advances in neural information processing systems. 2019 December, 285:3171–3180.

[9] Ren Y, Hu C, Tan X, et al. FastSpeech 2: Fast and High-Quality End-to-End Text to Speech. International Conference on Learning Representations; 2021 May 4; Vienna, Austria. 113:1127–1145.

[10] Kalchbrenner N, Elsen E, Simonyan K, Noury S, Casagrande N, Lockhart E, et al. Efficient neural audio synthesis. In: Dy JG, Krause A, editors. ICML’18: Proceedings of the 35th International Conference on Machine Learning; 2018 Jul 10-15; Stockholm, Sweden. 80: 2410–2419.

[11] Jemine C. Real-time-voice-cloning. Master’s Thesis, University of Liége. 2019.

[12] War V. Wikipedia, the free encyclopedia. Retrieved July. 2009;22: 2009.

[13] Centracchio F, Iemma U. An integrated approach to the direct simulation of brasses in the performance environment. Appl Acoust. 2021 Jun;177: 107935. doi: 10.1016/j.apacoust.2021.107935

[14] Byrd D, Saltzman E. The elastic phrase: Modeling the dynamics of boundary-adjacent lengthening. J Phon. 2003;31(2): 149–180. doi: 10.1016/S0095-4470(02)00085-2

[15] Alexander R, Sorensen T, Toutios A, Narayanan S. A modular architecture for articulatory synthesis from gestural specification. J Acoust Soc Am. 2019 May;146(6): 4458–4471. doi: 10.1121/1.5139413 31893678 PMC7043897

[16] Mullah HU. A comparative study of different text-to-speech synthesis techniques. Int J Sci Eng Res. 2015;6(6): 287–292.

[17] Bulyko I, Ostendorf M. Joint prosody prediction and unit selection for concatenative speech synthesis. Proceedings of the 2001 IEEE International Conference on Acoustics, Speech, and Signal Processing (Cat. No. 01CH37221); 2001 May 7-11; Salt Lake City, Utah. 781–784.

[18] Jeerapradit L, Suchato A, Punyabukkana P. HMM-based Thai singing voice synthesis system. Proceedings of the 2018 22nd International Computer Science and Engineering Conference (ICSEC); 2018 Nov 21—24; Thailand. 1–4.

[19] Saadi I, Mustafa A, Teller J, Farooq B, Cools M. Hidden Markov Model-based population synthesis. Transport Res B-Methodol. 2016 Aug;90: 1–21.

[20] Ping W, Peng K, Chen J. Clarinet: Parallel wave generation in end-to-end text-to-speech. arXiv preprint arXiv:1807.07281, 2018.

[21] Łańcucki A. Fastpitch: Parallel text-to-speech with pitch prediction. Proceedings of the 2021-2021 IEEE International Conference on Acoustics, Speech, and Signal Processing (ICASSP); 2021 June 6-11; Toronto, Canada. 6588–6592.

[22] Miao C, Shuang L, Liu Z, Minchuan C, Ma J, Wang S, et al. Efficienttts: An efficient and high-quality text-to-speech architecture. In: Meila M, Zhang T, editors. ICML’21: Proceedings of the 38th International Conference on Machine Learning; 2021 Jul 18-24; Jeju Island, South Korea. 139: 7700–7709.

[23] Jia Y, Zhang Y, Weiss R, Wang Q, Shen J, Ren F, et al. Transfer learning from speaker verification to multispeaker text-to-speech synthesis. Proceedings of the 32nd International Conference on Neural Information Processing Systems; 2018 Dec 3-8; Montréal, Canada. 31: 4485–4495.

